# Aging as a Risk Factor on the Immunoexpression of Pro-Inflammatory IL-1β, IL-6 and TNF-α Cytokines in Chronic Apical Periodontitis Lesions

**DOI:** 10.3390/biology11010014

**Published:** 2021-12-23

**Authors:** Quésia Euclides Teixeira, Dennis de Carvalho Ferreira, Alexandre Marques Paes da Silva, Lucio Souza Gonçalves, Fabio Ramoa Pires, Florence Carrouel, Denis Bourgeois, Irna Sufiawati, Luciana Armada

**Affiliations:** 1 Postgraduate Program in Dentistry, Estácio de Sá University, Rio de Janeiro 22790-710, Brazil; quesiaeuclides@yahoo.com (Q.E.T.); denniscf@gmail.com (D.d.C.F.); xandemps@hotmail.com (A.M.P.d.S.); luciogoncalves@yahoo.com.br (L.S.G.); ramoafop@yahoo.com (F.R.P.); 2 Health, Systemic, Process (P2S), UR 4129 Research Unit, University Claude Bernard Lyon 1, University of Lyon, 69008 Lyon, France; denis.bourgeois@univ-lyon1.fr; 3 Department of Oral Medicine, Universitas Padjadjaran, Bandung 40132, Indonesia; irna.sufiawati@fkg.unpad.ac.id

**Keywords:** apical periodontitis, IL-1β, IL-6, TNF-α, aging, endodontology, inflammation

## Abstract

**Simple Summary:**

Apical periodontitis often manifests as a chronic and asymptomatic disease. The formation of chronic apical periodontitis lesion (CAPL) occurs due an imbalance between microorganisms in a root canal system and a host’s immune response. The pro-inflammatory characteristic of periapical granulomas has been established; increased IL-1β, IL-6 and TNF-α has been documented. In humans over 65 years of age, the immune system becomes more fragile, including a persistent inflammatory response, making them more susceptible to reactivation of latent viruses, bacterial and viral pathogens, autoimmune diseases, opportunistic infections and neoplasms. The question of this study is as follows: in aging adults, do chronic apical periodontitis lesions as biomarkers have a risk effect on the immunoexpression of pro-inflammatory cytokines (IL-1β, IL-6 and TNF-α) compared to adults? The results demonstrated that the pro-inflammatory cytokines IL-1β, IL-6 and TNFα showed higher expression in the CAPL of the elderly than in the adult control group, thus suggesting that aging could be considered a modifier of periradicular disease. However, although aging is not the main cause for the development of CAPL, it seems to be able to influence its evolution. However, further elaborate research studies/analyses to elucidate the reasons and consequences of inflammation in the elderly are recommended.

**Abstract:**

Persistent inflammatory responses in the elderly may act as modifiers on the progression and repair of chronic apical periodontitis lesions (CAPLs). While the involvement of IL-1β, IL-6 and TNF-α in inflammatory responses and, particularly, in CAPL has been documented, their expression in elderly patients needs to be further characterized. Therefore, the purpose of this study was to evaluate and compare the expressions of pro-inflammatory cytokines in CAPL from elderly individuals with young/middle-aged individuals. Thirty CAPL (15 cysts and 15 granulomas) from elderly patients (>60 years) and 30 CAPL (15 cysts and 15 granuloma) from young/middle-aged individuals (20–56 years) were selected. Immunohistochemical reactions were performed against IL-1β, IL-6 and TNF-α. The slides were subdivided into five high-magnification fields and analyzed. The number of positive stains was evaluated for each antibody. There was no significant difference between the cytokines when the cysts and granuloma were compared in the two groups. In the young/middle-aged, only IL-1β showed a difference and was significantly higher in granulomas (*p* = 0.019). CAPL pro-inflammatory cytokine levels in the elderly were significantly higher than in young/middle-aged individuals (*p* < 0.05). The pro-inflammatory cytokines IL-1β, IL-6 and TNF-α were significantly higher in CAPL in the elderly compared with the young/middle-aged group. Further elaborate research studies/analyses to elucidate the reasons for and consequences of inflammation in the elderly are recommended.

## 1. Introduction

Apical periodontitis (AP) often manifests as a chronic and asymptomatic disease [1]. The formation of chronic apical periodontitis lesion (CAPL) occurs as a result of an imbalance between microorganisms (MOs) in a root canal system (RCS) and a host’s immune response. Bacteria can generate direct and indirect damage through the production of exotoxins, enzymes and metabolic products. This, in turn, is an inflammatory response of the host to a bacterial infection originating in the root canal system, which functions as an efficient barrier against the propagation of the infection to the alveolar bone and other regions of the body [2].

Several studies suggested that diseases might be a risk factor for endodontic outcomes [3,4,5,6]. These manifestations can evolve from asymptomatic symptoms to painful symptoms, causing delayed repair of some lesions and, in some cases, explain why well-treated canals result in failure [7]. Among the events that can interfere with the progress and even the cure of CAPL, we can highlight diabetes mellitus, genetic polymorphism, human immunodeficiency virus (HIV), smoking and osteoporosis [8,9]. Another factor that is considered by many to be a disease modifier is aging. Throughout life, the entire immune system undergoes morphological and functional changes that reach their functional peak at puberty, and then there is a gradual decline with aging. 

Although it was not possible to analyze the impact of increasing age in the prevalence and occurrence of AP, because the main part of the studies did not report age-stratified data, it appears that prevalence increases with increasing age, particularly in populations with low rates of dental extractions and accumulation of dental treatments, such as root canal therapy [10]. The prevalence of periapical radiolucency (PARL) and the prevalence of non-surgical root canal treatment (NSRCT) in the elderly were analyzed in a systematic review [11]. In the elderly, the prevalence of both NSRCT and PARL individually is increasing with age; while PARL in NSRCT teeth reduced with age. 

In humans over 65 years of age, it has been shown that, in this phase of life, there are many changes in the immune system that manifest as persistent inflammatory responses, causing an imbalance in the inflammatory process [12]. The immune function seems to go through a more significant change in individuals after reaching 60 years old [13]. Intrinsic factors related to aging (replicative stress, oxidative damage, genetic alterations, mitochondrial decline and cellular senescence) combine with extrinsic factors (dysbiosis of the oral microbiota and medication-related pathologies) to impair periodontal function.

Cytokines, such as TNF-α, interleukin-1 and -6, are implicated in the degradation of soft tissue, the promotion of lymphocytes and the resorption of bone directly or indirectly supporting osteoclastogenesis [14]. The pro-inflammatory characteristic of periapical granulomas has been established; increased IL-1β, IL-6 and TNF-α has been documented in symptomatic APs versus asymptomatic APs [15]. Nevertheless, the correlations of the levels of cytokines with the histological characteristics of these lesions were inconsistent [16]. Radicular cysts were characterized by a higher production of the pro-inflammatory cytokine IL-1β, as compared with periapical granulomas [17]. A positive correlation with IL-1β and IL-6 was also demonstrated, suggesting that stimulation loops involving pro-inflammatory cytokines could cause significant bone resorption at the apical periodontal zone [15]. The significance of the pro-inflammatory activity of IL-6 in the pathogenesis of apical periodontitis lesions is supported by the higher quantity of IL-6 in symptomatic lesions in comparison with asymptomatic lesions, as well as the control group [18]. Lastly, TNF-α has been reported as a major contributor of the bone loss [19].

Furthermore, there is proof that, when the immune system of the elderly is active, a high number of pro-inflammatory cytokines are released. “Inflammaging” is the term used to refer to this event in this phase of life when there are changes in the quantity and activity of immune cells; however, the mechanism that explains how or why these changes occur is still inconclusive [20]. The elevated level of pro-inflammatory cytokines IL-1β, IL-6, TNF-α, prostaglandins and anti-inflammatory mediators illustrates this immune system disorder in the elderly [12,21,22]. While studies in the older people have reported that IL-1β has shown no detectable age-related trend, IL-6, which is usually found at low concentrations in the blood, increases during aging [23]. With age, the pro-inflammatory cytokine TNF-α, a major actor in the immune response, increases and is implicated in age-related diseases [24].

In the elderly, the immune system becomes more fragile, making them more susceptible to reactivation of latent viruses, bacterial and viral pathogens, autoimmune diseases, opportunistic infections and neoplasms [25]. Among oral heterogeneous lesions, the first rank is chronic candidiasis and burning-mouth syndrome in the elderly, and aphthous stomatitis and acute candidiasis in the younger population [26].

However, the concept of “inflammaging” attributing the aging process to the pro-inflammatory context should not be dissociated from immunosenescence [27]. Actually, the term “immunosenescence” corresponds to a complete remodeling of the immune system and its microenvironment. This implies changes in both innate and adaptive components that appear with age [28]. Recent data are more in line with the interpretation that the process is a two-way relationship where immunosenescence is induced by inflammaging and vice versa [29].

Although some systematic reviews have sought to determine the prevalence of PA, none has looked at factors such as age that might alter its prevalence. The null hypothesis would be that there is no significant difference in risk effect on the immunoexpression of pro-inflammatory cytokines between the group of patients aged over 60 years and the group of young adults. In order to treat and prevent endodontic diseases in a more optimal way, it is necessary to know the periapical health status of the population [30].

Our research focused on the following topic: are established pro-inflammatory biomarkers relevant on the prevalence of CAPL in aging patients with endodontics treatment? The question of this study according to the principles of the PICO is as follows: in aging adults (population), does chronic apical periodontitis lesions as biomarkers (intervention) have a risk effect on the immunoexpression of pro-inflammatory cytokines (IL-1β, IL-6 and TNF-α) (outcome) compared to adults (control)?

## 2. Materials and Methods

### 2.1. Study Design and Ethical Approval

This study was a case control survey performed at the Dental School of the University Estácio de Sá, Brazil. This research was accepted by the Ethics Research Committee of Universidade Estácio de Sá (CAAE-47669715.2.0000.5284) and conducted under the principles of the declaration of Helsinki (2008).

### 2.2. Study Population

The study included 60 patients admitted to the Faculty of Dentistry between January 2017 and December 2019. Thirty CAPL (15 cysts and 15 granulomas) of patients over 60 years were assigned to the study group, and 30 CAPL (15 cysts and 15 granulomas) of patients between 20 to 56 years were allocated to the reference group for comparative analysis (ratio 1:1). All cases were obtained from the archives of the pathology laboratory of the Faculty of Dentistry of the Estácio de Sá University, and documentation with preceding medical records was involved. Lesions must be obtained through periradicular surgery. Exclusion criteria specimens included (i) undersize for histological processing; (ii) subjects with immune-suppressive diseases, such as auto-immune diseases, diabetes mellitus or acquired immune deficiency syndrome; and (iii) patients who have received of antibiotic, anti-inflammatory and/or analgesic medication one month prior to the surgery.

### 2.3. Sample Collection and Processing

The diagnosis of CAPL was confirmed through histopathological examination. The slides that were stained with eosin and hematoxylin were evaluated via two precalibrated evaluators, as a double model, using optical microscopy (Leica DM500, Heerbrugg, Sweden). The evaluation demonstrated that the cysts had a cavity completely or partially lined by epithelium and granulo-mas were composed by granulation tissue.

### 2.4. Histological Process

Histological sections were assembled on silanized microslides to perform the immunohistochemical tests, following a protocol described previously (13). The antibodies used were primary antibodies for IL-1β (1:100, rabbit, sc-7884), IL-6 (1:500, mouse, sc-130326) and TNF-α (1:50, mouse, sc-130349) from Santa Cruz Biotechnology (Dallas, TX, USA). The LSAB + HRP system (Dako K0690, DAKO North America, Carpinteria, CA, USA) was used as the secondary antibody. Liquid DAB (Liquid DAB + Substrate Chromogen System-Dako K3468, DAKO North America, Carpinteria, CA, USA) was used for staining the cytokine expression zones. For each antibody used, positive and negative controls (without primary antibody) were carried out according to the manufacturers’ instructions.

Images were observed by using optical microscopy (Leica DM500, Heerbrugg, Sweden). Each microslide was subdivided into 5 high-magnifications (40× microscopic magnification). The expression of each cytokine was assessed in the epithelium only for periradicular cysts and in the connective tissue. Cells that showed brownish staining at the cytoplasmic or membranous level were considered positive for cytokines.

### 2.5. Biomarker Analysis

To analyze the immunoexpression of each cytokine, values (0 to 2 for each field) were attributed to the number of positive markings for the antibodies. The scores given were according to a previously performed protocol [31]. Areas considered negative (0 points) were when there was no staining or when less than 5% of cells were positively stained, mild to moderate (1 point) was when 5–50% of cells were stained positively and severe (2 points) if over 50% of cells were considered positives. The slides were evaluated by each field and received scores, which together represent values from 0 (if all high-power fields tested were negative) to 10 (if all high-power fields tested were strongly positive). The final score is the cumulative value of the five fields, and the average of the five fields gives the immune-expression rate: negative (final average from 0 to 0.5), weak to moderate (0.6 to 1.2) and strong (1.3 to 2.0).

### 2.6. Statistical Analysis

Comparative data analysis was conducted with Graphpad Prism 6 for Windows (Graphpad Software, San Diego, CA, USA). As the studied variables (average of immunoexpression rate of the pro-inflammatory cytokines: IL-1β, IL-6, and TNF-α) were quantitative, the normality of the data was tested by using Kolmogorov–Smirnov and Shapiro–Wilk tests, and by graphical analysis. Thus, Mann–Whitney U test was used for the two groups’ comparisons (study group, elderly patients; control group, young/middle-aged) and between cyst and granuloma for each pro-inflammatory cytokine, whereas the Kruskal–Wallis test was applied for the comparisons among the three pro-inflammatory cytokines in each group (for cyst and granuloma). Descriptive data for variables were reported as mean ± standard deviation (SD). Statistical significance level was *p* < 0.05.

## 3. Results

### 3.1. Baseline Characteristics

Our study involved 30 cases and 30 controls, with a median (IQR) age of 69.0 (67.0–76.0) and 37.0 (32.0–47.0) years old, respectively. Table 1 summarizes the demographic and clinical data of the patients. Except for age, none of the parameters was statistically different between the two groups studied.

### 3.2. Biomarker Levels at Study Time Point

**Figure 1:**
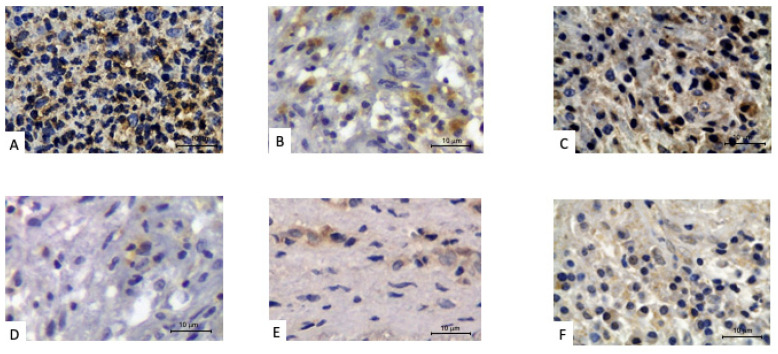
Periradicular granulomas with positive immunoperoxidase staining for pro-inflammatory cytokines. Older patient: (**A**) IL-1β, (**B**) IL-6 and (**C**) TNF-α. Younger/middle-age adult patient: (**D**) IL-1β, (**E**) IL-6 and (**F**) TNF-α.

**Figure 2:**
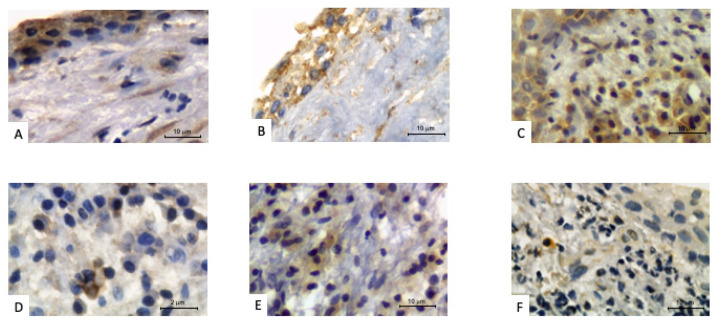
Periradicular cysts with positive immunoperoxidase staining for pro-inflammatory cytokines. Older patient: (**A**) IL-1β, (**B**) IL-6 and (**C**) TNF-α. Younger/middle-age adult patient: (**D**) IL-1β, (**E**) IL-6 and (**F**) TNF-α.

All the slides containing CAPL from elderly patients showed positive markings for the primary antibodies used. The results of the chemical mediator expressions in the images obtained by IHC (Figure 1A–C) in the periradicular granulomas of the elderly revealed that IL-1β was 16.6% focal, 16.6% weak/moderate and 66.8% strong; IL-6 was 67% weak/moderate and 33% strong; and TNF-α was: 83% weak/moderate and 17% strong. The results of the IHQ expression of these same chemical mediators in the images from the periradicular cysts of the elderly (Figure 2A–C) revealed that IL-1β was 40% weak/moderate and 60% strong; IL-6 was 20% focal, 20% weak/moderate and 60% strong; and TNF-α was 60% weak/moderate and 40% strong.

The numerical values of the means of the cytokine immunoexpressions in CAPL of the elderly are given in Table 2. No statistically significant difference was observed between the cytokine values in the cysts and granulomas. In the comparison of the pro-inflammatory cytokines between cysts and granulomas, there was also no significant difference.

Among youth/middle-aged of the control group, results of the expression of chemical mediators in the images obtained through IHC (Figure 1D–F) in periradicular granulomas revealed that IL-1β was 33.3% focal and 66.7% weak/moderate; IL-6 was 83.3% focal and 16.7% weak/moderate; and TNF-α was 83.3% focal and 16.7% weak/moderate. This same evaluation for the adult periradicular cysts (Figure 2D–F) revealed that IL-1β was 80% focal and 20% weak/moderate; IL-6 was 100% focal; and TNF-α was 60% focal and 40% weak/moderate.

The numerical values of the means of the cytokine immunoexpressions in the adult CAPL are given in Table 2. No statistically significant difference was found between cytokines in the cysts and granulomas. However, in the comparison of the pro-inflammatory cytokines between cyst and granulomas, there was a significant difference only in relation to IL-1β (*p* = 0.019), which showed a greater expression in granulomas.

**Figure 3:**
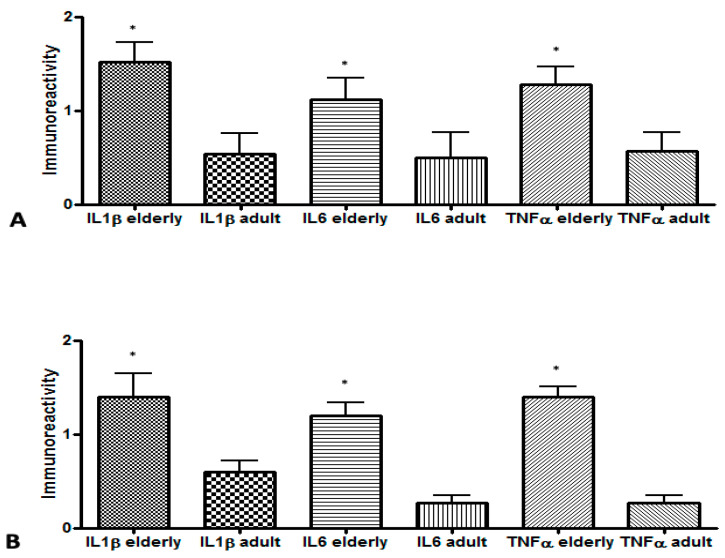
Comparison of pro-inflammatory cytokines in chronic apical periodontitis lesions between elderly and young/middle-aged patients: (**A**) cyst (IL-1β elderly–IL-1β adult, *p* = 0.008; IL-6 elderly–IL-6 adults, *p* = 0.016; TNF-α elderly–TNF-α adult; *p* = 0.008); (**B**) granuloma (IL-1β elderly–IL-1β adult, *p* = 0.041; IL-6 elderly–IL-6 adults, *p* = 0.002; TNF-α elderly–TNF-α adult; *p* = 0.002). Mann–Whitney test (* significant difference).

The comparison for each pro-inflammatory cytokine in CAPL (cyst and granuloma) between groups (Figure 3) revealed that the immunoexpression of the three cytokines studied was significantly higher in the elderly (study group) than in young/middle-aged patients (control group) (*p* < 0.05).

## 4. Discussion

Despite the fact that various authors have reported important modifications in the immune response of elderly patients, few studies have analyzed the impact of aging on the evolution and repair of CAPL. While the involvement of IL-1β, IL-6 and TNF-α in inflammatory reactions, and especially in CAPL, has been documented, their expression in elderly individuals needs to be further characterized. CAPL is a local inflammatory response caused by bacteria and their products inside the root canal, which appears to prevent the spread of this infection [32,33]. According to various reports in the literature, certain systemic factors may affect the development or cure of CAPL. Systemic factors—aging, smoking, comorbidities (diabetes mellitus, HIV and osteoporosis), disease modifiers (genetic polymorphisms), etc.—can have significant effects on the immune system and bone metabolism [9,34]. Studies in elderly rats [35,36] and in humans over the age of 65 [12] have shown that persistent inflammatory responses can occur in the elderly, due to an imbalance between the MOs and the host’s immune response. 

Various authors have reported that the elderly frequently have modifications in their immune systems that cause a marked release of blood pro-inflammatory cytokines (i.e., inflammaging) [20]. The findings of the present study, where there was a significant increase in IL-1β, IL-6 and TNF-α in the CAPL of elderly individuals, are in line with such observations. Although it is difficult to draw a significant conclusion to answer the global question based on three cytokine markers, these three pro-inflammatory cytokines are among the most studied in the literature. Their role in inflammation is highlighted. Thus, when the immune system of the elderly is active, a high number of IL-1 β, IL-6 and TNF- α are released [21,22]. The results of this study are also consistent with those of another published study [34], where it was possible to observe a similar growth in the production of TNF-α, IL-1β and IL-6 in older (elderly) rats in comparison with the younger.

According to various authors, the aging process affects the immune system [37,38,39,40], leading to increased levels of cytokines [12,20]. Furthermore, the excessive production of these cytokines could well result in the emergence of chronic diseases. These changes cause the elderly’s immune system to become more fragile, and therefore the reactivation of latent viruses, bacterial and viral pathogens, autoimmune diseases, opportunistic infections and neoplasms become more likely [25]. There are reports in the literature stating that an increased production of TNF-α, which inhibits the differentiation of osteoblasts and stimulates the production of RANKL, contributes to less bone formation in elderly animals and thus contributes to senile osteoporosis. These changes that can lead to resorption are also triggered by the immunological changes caused by aging [39]. 

Tissue homeostasis is disrupted in any inflammatory process, and this process involved both recruitment and retention of the cells of hematopoietic origin that go to the site affected to start the tissue repair process. IL-1 β is one of the cytokines that activate the inflammatory process; however, if released in an unregulated manner, it can lead to the development of chronic inflammatory conditions or the aggravation of the process [41]. The fact that elderly individuals had significantly higher values (*p* = 0.0034) of IL-1β compared to young/middle-aged individuals may be due to the phenomenon described above (i.e., inflammaging), where the immune system of the elderly acts in a disorderly manner, leading to a greater release of IL-1β in the cysts of the elderly. In addition, inflammaging may also be the reason for a higher presence of IL-6 in granulomas of elderly individuals in comparison with adult ones (*p* = 0.0019). 

Some cells, such as T-lymphocytes and macrophages, play a key role in this immune response; furthermore, these cells are involved in the release of some cytokines [42,43]. Other authors determined the presence of macrophages in cysts and granulomas of middle-aged and elderly patients with specific markers and found a lower expression of macrophages in the older patients. This result suggests that elderly patients are more likely to suffer endodontic treatment failure [44]. 

According to the literature, cysts originate from periapical granulomas, which form from endodontic infections that have remained in place for a long period of time [45,46]. When no measures are taken to treat the endodontic infection or in cases of treatment failure where CAPL persists at the site with no reduction of the inflammatory condition, the epithelial remnants of Malassez present inside the granulomas may, over time, induce the formation of epithelial tissue. The formation of this epithelial tissue is induced by bacterial endotoxins or inflammatory cytokines of the host [38,47,48]. The formation of epithelium inside the root cysts prevents the penetration of and the survival of macrophages, among other cells of the host’s immune system, inside the cysts. This process may also explain why, in the present study, adult patients had significantly higher levels of IL-1β in granulomas (*p* = 0.0019). Another study that analyzed 80 adult patients (18–55 years old) with CAPL demonstrated that increased levels of interleukin-1β were associated with chronic lesions in the acute exacerbation stages and granulomas in the initial stages of development. However, persistent granulomas, non-inflamed cysts, recently endodontically treated teeth and healed tissues showed significantly lower levels of interleukin-1β. These authors concluded that the differences in the amount of interleukin-1β correlated with the progression of the lesion and stages of development [49]. 

Previous studies showed that the presence of cells associated with the host’s immune response, such as mast cells, are greater in granulomas than in periapical cysts [50,51]. These cells are involved in the release of chemical mediators, such as TNF-α and IL-1 β. In our study, there was a greater expression of IL-1β in granulomas only in the adult patients. The presence of TNF-α in high amounts can result in vascular and inflammatory changes in the area of the lesion, which can, consequently, lead to a further increase in TNF-α [50]. Our results showed TNF-α was present in greater quantities (*p* = 0.001) in elderly individuals, thus suggesting that their immune response may result in an increase in CAPL and, consequently, increase failure rates in endodontic treatments. Macrophages, mononuclear phagocytes and monocytes are the most important stimuli to activate the synthesis of pro-inflammatory cytokines. However, in the meantime, since individuals over the age of 60 years old (probably) have an altered immune system, they should be treated in a way that this particularity is taken into consideration.

One of the limitations of the study was the small number of samples, which enables us to consider the results obtained as intermediate; thus, in the future, a subsequent study is recommended. A larger sample size would have reduced the age range of the younger group by creating an additional age subcategory—young adult, adult and senior. As a retrospective study, with the necessary caution in the causal interpretation of the observed associations, this study may have some data bias. It would have been interesting to include dynamic data of CAPL that are not available in the medical data record, such as active phase and duration.

This study, for methodological considerations, did not investigate associations between cytokine expression and clinical/imaging manifestations of AP, despite the fact that clarification on lesion size relative to cytokine expression would have been useful to assess the expression levels of TNF-α. Likewise, our research really only performed one type of assessment and did not back up the data with any other type of experiment, such as qRT-PCR from the lesion, RNA-seq or identification of cell types in the lesion itself. Finally, it should be noted that our study presents a selection gender bias with an overrepresentation of men that must be must be considered, as the age-related changes in several immunological parameters are different between genders, probably due to women’s lower biological age [52].

## 5. Conclusions

The results demonstrated that the pro-inflammatory cytokines IL-1β, IL-6 and TNFα showed higher expression in the CAPL of the elderly than in the adult control group, which suggests that aging could be considered a modifier of periradicular disease. However, although aging is not the main cause for the development of CAPL, it seems to be able to influence its evolution. However, further elaborate research studies/analyses to elucidate the reasons and consequences of inflammation in the elderly are recommended.

## Figures and Tables

**Table 1:** Demographic and clinical characteristics of young/middle-aged and elderly patients.

Parameter	Elderly	Young/Middle-Aged	*p*-Value
Age ^1^	70.4 ± 5.34	39.9 ± 9.3	<0.00001
Gender ^2^	19 females 11 males	18 females 12 males	1.0
Localization ^2^	18 anterior 12 posterior	19 anterior 11 posterior	1.0
Anatomical location ^2^	17 maxilla 13 mandible	20 maxilla 10 mandible	0.59

^1^ Mann–Whitney test was used to compare age (mean ± standard deviation). ^2^ Fisher’s exact test was used to compare gender, localization and anatomical location. Statistical significance (*p* < 0.05).

**Table 2:** Comparative analysis of the expressions of the pro-inflammatory cytokines in granulomas and apical cysts of young/middle-aged and elderly patients.

Cytokine	Groups					
	Elderly			Young/Middle-Aged		
	Cysts ^1^	Granulomas ^1^	*p*-Value ^2^	Cysts ^1^	Granulomas ^1^	*p*-Value ^2^
IL-1β	1.52 ± 0.46	1.40 ± 0.59	0.792	0.32 ± 0.17	0.60 ± 0.26	0.019
IL-6	1.12 ± 0.50	1.20 ± 0.33	0.930	0.24 ± 0.16	0.26 ± 0.20	0.937
TNF-α	1.28 ± 0.41	1.40 ± 0.25	0.662	0.40 ± 0.26	0.26 ± 0.20	0.536
*p*-value ^3^	0.500	0.420		0.690	0.060	

^1^ Values are expressed as mean ± standard deviation of the scores given for staining intensity. ^2^ Mann–Whitney test. ^3^ Kruskal–Wallis test.

## Data Availability

The data presented in this study are available upon request from the corresponding author.

## References

[1] Tibúrcio-Machado C.S., Michelon C., Zanatta F.B., Gomes M.S., Marin J.A., Bier C.A. (2021). The Global Prevalence of Apical Periodontitis: A Systematic Review and Meta-Analysis. Int. Endod. J..

[2] Ricucci D., Lopes W.S.P., Loghin S., Rôças I.N., Siqueira J.F. (2018). Large Bacterial Floc Causing an Independent Extraradicular Infection and Posttreatment Apical Periodontitis: A Case Report. J. Endod..

[3] Laukkanen E., Vehkalahti M.M., Kotiranta A.K. (2019). Impact of Systemic Diseases and Tooth-Based Factors on Outcome of Root Canal Treatment. Int. Endod. J..

[4] Aminoshariae A., Kulild J.C., Mickel A., Fouad A.F. (2017). Association between Systemic Diseases and Endodontic Outcome: A Systematic Review. J. Endod..

[5] Naqvi A.R., Shango J., Seal A., Shukla D., Nares S. (2018). Herpesviruses and MicroRNAs: New Pathogenesis Factors in Oral Infection and Disease?. Front. Immunol..

[6] Hernández Vigueras S., Donoso Zúñiga M., Jané-Salas E., Salazar Navarrete L., Segura-Egea J.J., Velasco-Ortega E., López-López J. (2016). Viruses in Pulp and Periapical Inflammation: A Review. Odontology.

[7] Karamifar K., Tondari A., Saghiri M.A. (2020). Endodontic Periapical Lesion: An Overview on the Etiology, Diagnosis and Current Treatment Modalities. Eur. Endod. J..

[8] Segura-Egea J.J., Jiménez-Pinzón A., Ríos-Santos J.V., Velasco-Ortega E., Cisneros-Cabello R., Poyato-Ferrera M.M. (2008). High Prevalence of Apical Periodontitis amongst Smokers in a Sample of Spanish Adults. Int. Endod. J..

[9] Segura-Egea J.-J., Castellanos-Cosano L., Machuca G., López-López J., Martín-González J., Velasco-Ortega E., Sánchez-Domínguez B., López-Frías F.-J. (2012). Diabetes Mellitus, Periapical Inflammation and Endodontic Treatment Outcome. Med. Oral Patol. Oral Cir. Bucal.

[10] Bjørndal L., Kirkevang L.-L., Whitworth J.M. (2018). Textbook of Endodontology.

[11] Hamedy R., Shakiba B., Pak J.G., Barbizam J.V., Ogawa R.S., White S.N. (2016). Prevalence of Root Canal Treatment and Periapical Radiolucency in Elders: A Systematic Review. Gerodontology.

[12] Franceschi C., Bonafè M., Valensin S., Olivieri F., De Luca M., Ottaviani E., De Benedictis G. (2000). Inflamm-Aging: An Evolutionary Perspective on Immunosenescence. Ann. N. Y. Acad. Sci..

[13] Fuentes E., Fuentes M., Alarcón M., Palomo I. (2017). Immune System Dysfunction in the Elderly. An. Acad. Bras. Cienc..

[14] Fouad A.F., Khan A.A., Silva R.M., Kang M.K. (2020). Genetic and Epigenetic Characterization of Pulpal and Periapical Inflammation. Front. Physiol..

[15] Jakovljevic A., Knezevic A., Karalic D., Soldatovic I., Popovic B., Milasin J., Andric M. (2015). Pro-Inflammatory Cytokine Levels in Human Apical Periodontitis: Correlation with Clinical and Histological Findings. Aust. Endod. J..

[16] Teixeira-Salum T.B., Rodrigues D.B.R., Gervásio A.M., Souza C.J.A., Rodrigues V., Loyola A.M. (2010). Distinct Th1, Th2 and Treg Cytokines Balance in Chronic Periapical Granulomas and Radicular Cysts. J. Oral Pathol. Med..

[17] Dessaune Neto N., Porpino M.T.M., Antunes H.D.S., Rodrigues R.C.V., Perez A.R., Pires F.R., Siqueira J.F., Armada L. (2018). Pro-Inflammatory and Anti-Inflammatory Cytokine Expression in Post-Treatment Apical Periodontitis. J. Appl. Oral Sci..

[18] Azuma M.M., Samuel R.O., Gomes-Filho J.E., Dezan-Junior E., Cintra L.T.A. (2014). The Role of IL-6 on Apical Periodontitis: A Systematic Review. Int. Endod. J..

[19] Kitaura H., Kimura K., Ishida M., Kohara H., Yoshimatsu M., Takano-Yamamoto T. (2013). Immunological Reaction in TNF-α-Mediated Osteoclast Formation and Bone Resorption in Vitro and in Vivo. Clin. Dev. Immunol.

[20] Shaw A.C., Goldstein D.R., Montgomery R.R. (2013). Age-Dependent Dysregulation of Innate Immunity. Nat. Rev. Immunol..

[21] Chung H.Y., Kim D.H., Lee E.K., Chung K.W., Chung S., Lee B., Seo A.Y., Chung J.H., Jung Y.S., Im E. (2019). Redefining Chronic Inflammation in Aging and Age-Related Diseases: Proposal of the Senoinflammation Concept. Aging Dis..

[22] Michaud M., Balardy L., Moulis G., Gaudin C., Peyrot C., Vellas B., Cesari M., Nourhashemi F. (2013). Proinflammatory Cytokines, Aging, and Age-Related Diseases. J. Am. Med. Dir. Assoc..

[23] Nikolich-Žugich J. (2018). The Twilight of Immunity: Emerging Concepts in Aging of the Immune System. Nat. Immunol..

[24] Rea I.M., Gibson D.S., McGilligan V., McNerlan S.E., Alexander H.D., Ross O.A. (2018). Age and Age-Related Diseases: Role of Inflammation Triggers and Cytokines. Front. Immunol..

[25] Linton P.J., Dorshkind K. (2004). Age-Related Changes in Lymphocyte Development and Function. Nat. Immunol..

[26] Popa C., Filioreanu A.M., Stelea C., Maftei G.A., Popescu E. (2018). Prevalence of Oral Lesions Modulated by Patient’s Age: The Young versus the Elderly. Rom. J. Oral Rehabil..

[27] Appay V., Sauce D. (2017). Assessing Immune Aging in HIV-Infected Patients. Virulence.

[28] Cunha L.L., Perazzio S.F., Azzi J., Cravedi P., Riella L.V. (2020). Remodeling of the Immune Response with Aging: Immunosenescence and Its Potential Impact on COVID-19 Immune Response. Front. Immunol..

[29] Fulop T., Witkowski J.M., Olivieri F., Larbi A. (2018). The Integration of Inflammaging in Age-Related Diseases. Semin. Immunol..

[30] Vos T., Abajobir A.A., Abate K.H., Abbafati C., Abbas K.M., Abd-Allah F., Abdulkader R.S., Abdulle A.M., Abebo T.A., Abera S.F. (2017). GBD 2016 Disease and Injury Incidence and Prevalence Collaborators Global, Regional, and National Incidence, Prevalence, and Years Lived with Disability for 328 Diseases and Injuries for 195 Countries, 1990–2016: A Systematic Analysis for the Global Burden of Disease Study 2016. Lancet.

[31] Ajuz N.C., Antunes H., Mendonça T.A., Pires F.R., Siqueira J.F., Armada L. (2014). Immunoexpression of Interleukin 17 in Apical Periodontitis Lesions. J. Endod..

[32] Sasaki H., Hirai K., Martins C.M., Furusho H., Battaglino R., Hashimoto K. (2016). Interrelationship Between Periapical Lesion and Systemic Metabolic Disorders. Curr. Pharm. Des..

[33] Siqueira J.F., Rôças I.N. (2008). Clinical Implications and Microbiology of Bacterial Persistence after Treatment Procedures. J. Endod..

[34] Kovacs E.J., Palmer J.L., Fortin C.F., Fülöp T., Goldstein D.R., Linton P.-J. (2009). Aging and Innate Immunity in the Mouse: Impact of Intrinsic and Extrinsic Factors. Trends Immunol..

[35] Chelvarajan R.L., Liu Y., Popa D., Getchell M.L., Getchell T.V., Stromberg A.J., Bondada S. (2006). Molecular Basis of Age-Associated Cytokine Dysregulation in LPS-Stimulated Macrophages. J. Leukoc. Biol..

[36] Singh S., Sharma N., Upadhyay C., Kumar S., Rathi B. (2019). Small Molecules Effective against Liver and Blood Stage Malarial Infection. Curr. Top. Med. Chem..

[37] Duque G., Troen B.R. (2008). Understanding the Mechanisms of Senile Osteoporosis: New Facts for a Major Geriatric Syndrome. J. Am. Geriatr. Soc..

[38] Lin J.T., Lane J.M. (2006). Rehabilitation of the Older Adult with an Osteoporosis-Related Fracture. Clin. Geriatr. Med..

[39] Pietschmann P., Skalicky M., Kneissel M., Rauner M., Hofbauer G., Stupphann D., Viidik A. (2007). Bone Structure and Metabolism in a Rodent Model of Male Senile Osteoporosis. Exp. Gerontol..

[40] Raisz L.G. (2005). Pathogenesis of Osteoporosis: Concepts, Conflicts, and Prospects. J. Clin. Investig..

[41] Di Paolo N.C., Shayakhmetov D.M. (2016). Interleukin 1α and the Inflammatory Process. Nat. Immunol..

[42] Langeland K., Block R.M., Grossman L.I. (1977). A Histopathologic and Histobacteriologic Study of 35 Periapical Endodontic Surgical Specimens. J. Endod..

[43] Martinho F.C., Leite F.R.M., Nascimento G.G., Cirelli J.A., Gomes B.P.F.A. (2014). Clinical Investigation of Bacterial Species and Endotoxin in Endodontic Infection and Evaluation of Root Canal Content Activity against Macrophages by Cytokine Production. Clin. Oral Investig..

[44] de Almeida N.F., Brasil S.D., Ferreira D.D., Armada L. (2017). Aging Effects in the Expression of Macrophages in Post-Treatment Apical Periodontitis Lesions. Spec. Care Dent..

[45] Meghji S., Qureshi W., Henderson B., Harris M. (1996). The Role of Endotoxin and Cytokines in the Pathogenesis of Odontogenic Cysts. Arch. Oral Biol..

[46] Takahashi K. (1998). Microbiological, Pathological, Inflammatory, Immunological and Molecular Biological Aspects of Periradicular Disease. Int. Endod. J..

[47] de Andrade A.L., Nonaka C.F., Gordón-Núñez M.A., de Almeida Freitas R., Galvão H.C. (2013). Immunoexpression of Interleukin 17, Transforming Growth Factor Β1, and Forkhead Box P3 in Periapical Granulomas, Radicular Cysts, and Residual Radicular Cysts. J. Endod..

[48] Marçal J.R.B., Samuel R.O., Fernandes D., de Araujo M.S., Napimoga M.H., Pereira S.A.L., Clemente-Napimoga J.T., Alves P.M., Mattar R., Rodrigues V. (2010). T-Helper Cell Type 17/Regulatory T-Cell Immunoregulatory Balance in Human Radicular Cysts and Periapical Granulomas. J. Endod..

[49] Popovska L., Dimova C., Evrosimoska B., Stojanovska V., Muratovska I., Ćetenović B., Marković D. (2017). Relationship between IL-1β Production and Endodontic Status of Human Periapical Lesions. Vojnosanit. Pregl..

[50] de Oliveira Rodini C., Batista A.C., Lara V.S. (2004). Comparative Immunohistochemical Study of the Presence of Mast Cells in Apical Granulomas and Periapical Cysts: Possible Role of Mast Cells in the Course of Human Periapical Lesions. Oral Surg. Oral Med. Oral Pathol. Oral Radiol. Endod..

[51] Patidar K.A., Parwani R.N., Wanjari S.P., Patidar A.P. (2012). Mast Cells in Human Odontogenic Cysts. Biotechnic Histochem..

[52] Caruso C., Accardi G., Virruso C., Candore G. (2013). Sex, Gender and Immunosenescence: A Key to Understand the Different Lifespan between Men and Women?. Immun. Ageing.

